# Analysis of IS*6110* insertion sites provide a glimpse into genome evolution of *Mycobacterium tuberculosis*

**DOI:** 10.1038/srep12567

**Published:** 2015-07-28

**Authors:** Tanmoy Roychowdhury, Saurav Mandal, Alok Bhattacharya

**Affiliations:** 1 School of Computational and Integrative Sciences, Jawaharlal Nehru University, New Delhi; 2 School of Life Sciences, Jawaharlal Nehru University, New Delhi.

## Abstract

Insertion sequence (IS) *6110* is found at multiple sites in the *Mycobacterium tuberculosis* genome and displays a high degree of polymorphism with respect to copy number and insertion sites. Therefore, IS*6110* is considered to be a useful molecular marker for diagnosis and strain typing of *M. tuberculosis*. Generally IS*6110* elements are identified using experimental methods, useful for analysis of a limited number of isolates. Since short read genome sequences generated using next-generation sequencing (NGS) platforms are available for a large number of isolates, a computational pipeline for identification of IS*6110* elements from these datasets was developed. This study shows results from analysis of NGS data of 1377 *M. tuberculosis* isolates. These isolates represent all seven major global lineages of *M. tuberculosis*. Lineage specific copy number patterns and preferential insertion regions were observed. Intra-lineage differences were further analyzed for identifying spoligotype specific variations. Copy number distribution and preferential locations of IS*6110* in different lineages imply independent evolution of IS*6110*, governed mainly through ancestral insertion, fitness (gene truncation, promoter activity) and recombinational loss of some copies. A phylogenetic tree based on IS*6110* insertion data of different isolates was constructed in order to understand genome level variations of different markers across different lineages.

*Mycobacterium tuberculosis*, the etiologic agent of tuberculosis, causes large scale morbidity and mortality particularly in developing countries. The bacterium has shown significant increase in its ability to become drug-resistant, creating a major public health crisis^1^. Analysis of *M. tuberculosis* genome evolution may help us to better understand the genotype-phenotype relationship in this organism. Different strain typing methods have revealed world-wide diversity of *M.tuberculosis*. Large sequence polymorphisms (LSPs) have helped to distinguish six geographically restricted major global lineages^2^ of this pathogen known as Indo-Oceanic (L1), East Asian (L2), Indian-East African (L3) Euro-American (L4) and West African I & II (L5, L6). Further, L1, L5 and L6 strains have been classified as ancient strains whereas other three lineages have been termed modern based on a deletion known as TbD1^3^. Later, another distinct phylogenetic lineage, L7 was identified in Ethiopia^4^. Single nucleotide polymorphisms (SNPs) were also shown to be consistent with this classification^5^. Other popular strain typing methods are based on variable number of tandem repeats (VNTR) and presence/absence of spacer oligonucleotide known as spoligotyping^6^. Spoligotyping results are also consistent^7^ with lineage-based classification as spoligotype Beijing and CAS represent L2 and L3 lineages respectively where LAM, T, X, S and Haarlem strains make up the L4 lineage. Lineage-based classification is thought to reflect observed variability in emergence of drug-resistance^8^, immune response^9^ and disease severity^10^. Results from different studies indicate that *M. tuberculosis* undergoes clonal evolution governed mainly by genetic drift^11^.

Insertion sequences (IS) make up a major component of bacterial repetitive elements and these have often been used for species and strain typing^12^. IS*6110* is specific to the *M. tuberculosis* complex and can be used for diagnosis, that is, the presence of *M. tuberculosis* cells in a biological sample. Since these elements are mobile and are located at different sites, IS*6110* based restriction fragment length polymorphism (RFLP) has become a popular tool for strain typing^13^,^14^,^15^. One limitation of this approach is that not all isolates display multiple copies of these elements and some lack even a single copy^16^,^17^. Accordingly, *M. tuberculosis* strains are frequently classified into high IS copy-number (>7) and low IS copy-number strains^18^. It is not clear if these two groups of organisms show different physiological or pathogenic behavior. Although it is believed that high copy number of IS*6110* in highly pathogenic strains (Beijing) provides a selective advantage, drug resistance and outbreaks have also been associated with low copy number strains^19^. Evolutionary models that explain the control of IS*6110* copy number have been developed^20^. Frequently IS*6110* elements are found inserted in a 36-bp array known as Direct Repeat region (DR region: Rv2813-Rv2820c, RD207)^21^. Almost all *M. tuberculosis* complex (MTBC) isolates have an IS*6110* element in the DR region and is considered to be the original insertion site in MTBC genome^19^. Analysis of IS*6110* insertion sites demonstrated a lack of sequence context specificity for integration, though several insertion cold spots and hotspots have been identified^22^,^23^. Some of the insertion hotspots are intergenic, e.g. Rv0001-Rv0002, but many are intragenic; Rv0797 (IS1547 transposase), Rv1755c (*plcD*), Rv1758 (*cut1*), Rv1777 (*cyp144*), Rv2351 (*plcA*), Rv3183, Rv3327 and many PE-PPE family proteins. Alonso *et al.*^21^ found preferential insertion locations in the Beijing strains in comparison to the non-Beijing strains of *M. tuberculosis*. Analysis of modern strains revealed ancestral/inherited insertion sites rather than insertion hotspots^24^.

IS*6110* insertion in any intragenic region may have a deleterious as well as a selective outcome. Generally genes involved in virulence, information pathway, lipid metabolism and cell wall synthesis are not preferred targets of transposition^23^. On the other hand the maximum number of transposition is found in multi-gene families^25^, such as the PPE gene family, because phenotypic effects can be masked by other copies. Moreover, PPE genes are thought to act as a variable surface antigen^26^ and their disruption can be beneficial for immuno-evasion. Insertion of IS*6110* in the *plcD* gene is related to extrathoracic disease^27^. Mycobacterial drug resistance is also shown to be associated with insertion events, for example, IS*6110* insertions in the *tlyA* gene is observed in capreomycin resistant *M. smegmatis* and *M. tuberculosis*^28^; isoniazid, ethionamide and para-aminosalicylic acid resistance of *M. bovis* has been associated with insertion in *katG*, *etaA* and *thyA* genes respectively^29^,^30^. Some IS*6110* intragenic mutants were also shown to have increased virulence as demonstrated by survival time of infected mice^31^. High frequency of SNPs, expansion/contraction of tandem repeats and larger genomic deletions have also been reported in regions flanking IS*6110*^22^. LSPs can occur due to recombination of two neighboring copies of IS*6110*. These events can be detected by the absence of 3–4 bp direct repeats in surrounding regions. Homologous recombination plays a major role in deletion particularly near the ipl locus with high occurrence of IS*6110*^32^. IS*6110* carries an outward directed promoter at its 3′ end, thus the element can act as a mobile promoter^33^. This promoter is able to up-regulate several downstream genes^34^. Soto *et al.* showed increased virulence of *M. tuberculosis* by IS*6110* insertion upstream of the *phoP* gene^35^, a transcriptional regulator important for bacterial growth. Up-regulation of *phoP* by an IS*6110* located 75 bp upstream of the gene in a multi-drug resistant *M. tuberculosis* was found in isolates during an outbreak in Spain^35^. Overall, IS*6110* transposition is important for evolution of *M. tuberculosis* genome and consequently alteration in the physiology and pathogenesis of the organism.

A number of methods have been used for mapping IS*6110* distribution. Some of these are electrophoresis based IS*6110*-RFLP^36^, PCR based fluorescent amplified fragment length polymorphism (fAFLP)^24^, IS*6110* 5′ and 3′ fluorescent polymorphism (IS*6110*-5′3′FP)^37^, DNA microarray based SiteMapping^38^ and targeted amplification of IS elements followed by sequencing or IS-seq^39^. All these methods have been useful in identification of many IS insertion sites. Unfortunately due to bias in amplification it is likely that many sites may still be missed^38^. Computational identification of IS insertion sites from whole genome sequences offers an alternate approach that appears to yield relatively complete information. Moreover, with the availability of NGS datasets, it is now possible and easier to identify these elements from large number of isolates that allow better understanding of their role in evolution of *M. tuberculosis* genome. Since most of the NGS genome data is available in an unassembled form, methods that can use these data are going to be useful. A computational pipeline was used to identify the positions of IS elements in *M. tuberculosis* genome from unassembled NGS data^40^. This method, in principle, can be used to analyze IS elements of any organism. Overall, this study provides analysis of 1377 publicly available NGS datasets of *M. tuberculosis* isolates to generate a global picture of IS*6110* distribution.

## Results & Discussion

### Identification of IS elements from NGS data

NGS Data used for this study is described in Table 1. The number of sequenced isolates varied for different lineages, for example, there were only 4 sequences from L7 while 666 sequences could be accessed from the L4 lineage. The pipeline described here used a split-read approach to identify the junction of IS*6110* and flanking sequences from NGS reads. Since the datasets were unassembled, the positions of the insertion sites in isolates were defined using the reference genome of *M. tuberculosis* H37Rv. The validity of the pipeline was checked using simulated NGS data of H37Rv and computationally identified and experimentally verified IS*6110* insertion sites^40^. In an earlier study a PCR based approach could not validate all predictions. Absence of PCR products is quite often due to number of factors that include low amount of clinical samples, quality of DNA extracted from patient tissues, extensive secondary structure and or nature of DNA sequence besides absence of target sequences. It is not possible to experimentally validate the identified sites in isolates that are part of this study as data have been generated in large number by different laboratories around the World using patient isolates. However, some of the sites identified by us are already part of a number of published reports.

**Figure 1:**
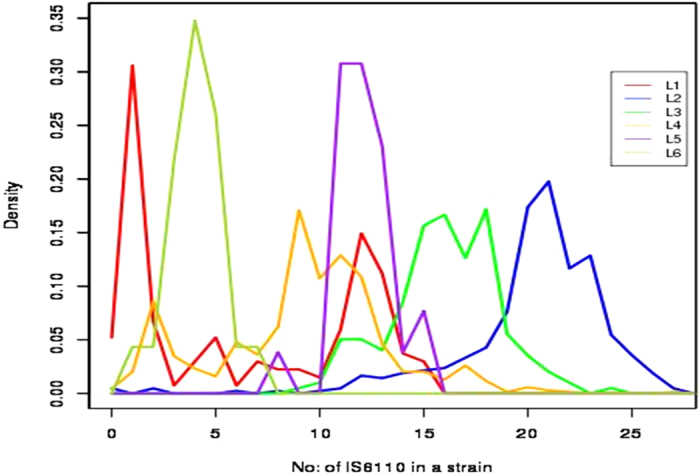
Lineage based IS*6110* copy number distribution in six major global lineages of *M.* *tuberculosis.* Each lineage is represented by a different color as mentioned in the box.

The analysis showed that not all isolates of *M. tuberculosis* carry IS*6110*. A total of 12 isolates from L1 (6.03%), L2 (0.53%) and L4 (0.45%) were part of this group. The number of copies of this element varied from 0 to 27 across all isolates (supplementary figure 1). Lineage based distribution of IS*6110* copy number was analyzed in order to understand the relationship between lineages and IS*6110* expansion. All four L7 isolates displayed only one copy of the element. Density distribution of IS*6110* copy number in the other six lineages is shown in Fig. 1. The results showed inter and intra-lineage variations. Isolates belonging to L2 lineage displayed highest mean copy number 20.24 as compared to mean copy number 16.13 observed among L3 isolates. Statistical analysis of IS*6110* copy number among the different isolates showed significant differences among the different lineages (One-way Anova, F_(6,1370)_ = 384.36, P < 0.01). However, not all lineages showed a similar pattern. L1 and L4 displayed large variations within their respective groups (supplementary table 1) and this is in agreement with SNP data^1^. A bimodal distribution was observed for L1 isolates (Fig. 1). L1 isolates could be classified in two major categories; one group with 1–2 copies and another with 12–13 copies (see next section for spoligotype based classification). In spite of the large variation within L1 and L4, very high copy numbers (>20) were rarely seen. No relationship between copy number and sequence conservation of the element in different lineages was observed. For example, two West African lineages L5 and L6 display a highly similar nucleotide sequence but different IS copy numbers (supplementary table 1). These results suggest that the expansion of the IS*6110* element may have taken place independently in each lineage. It was hypothesized that IS*6110* copy number variation largely depends on insertion of IS*6110* in transcriptionally active/inactive regions of the genome^41^. This could result in high intra-lineage variability and low inter-lineage differences (as some of the strains from each lineage may become high/low copy number), but this was not observed in present study.

### Lineage specific insertions

This analysis did not reveal the existence of general hotspots for insertions in different lineages except the DR region where an IS*6110* can be found in 99.26% isolates. However, this study did reveal the presence of preferential insertion regions (PIR) in each lineage. General hotspots signify repeated independent insertion into the same spot whereas PIRs are considered as one inherited insertion passed down through a lineage. In order to recognize lineage specific PIRs, H37Rv genome sequence was divided into non-overlapping bins of 100 bp. Presence of IS*6110* in >80% strains in a genomic bin of a specific lineage and <10% of isolates in all other lineage, was considered as a criterion for PIR. Lineages L2, L3, L5 and L6 displayed a high level of conservation in PIRs and the number of conserved sites was found to be positively correlated with the number of copies present in a given lineage (Pearson correlation coefficient, R = 0.895, P < 0.05). High copy number isolates of L2 were found with 10 such locations. Alonso *et al.*^21^ reported preferential insertion locations in Beijing strains (L2), this study filtered out locations which are rare in other lineages, such as, Rv0001-Rv0002 (intergenic), Rv1371 and *idsB*. The results presented here with respect to L3 isolates (6 PIRs) have not been reported before. Most conserved sites mapped to Rv0395, Rv1504c and Rv3845-Rv3846 (intergenic). Isolates belonging to L1 and L4 lineages showed much higher intra-lineage variations and lineage specific PIRs were not observed. These isolates were further analyzed. Table 2 lists PIRs found in isolates of different lineages. These locations can be used as probable molecular markers for identification of specific isolates of each lineage.

Each lineage can be further subdivided into several spoligotyped classes. For any genomic bin, presence of IS*6110* in >75% strains of a specific spoligotype and <20% in all other spoligotype groups of the same lineage, was considered as a threshold for identification of spoligotype specific PIR. Ninety isolates from the L1 lineage were available with spoligotype information. While high copy number isolates belong to EAI2 or EAI6, low copy number isolates are of EAI1, EAI3 or EAI4 spoligotypes (supplementary table 2). Among these, high copy number isolates of EAI2 and EAI6 displayed conservation of PIRs whereas low copy number isolates did not contain any PIR (Table 3). The spoligotype EAI5 group of isolates showed a high degree of variability in terms of copy number (supplementary table 2). Similarly, 606 isolates belonging to the L4 lineage were analyzed with respect to their spoligotype pattern. Only low copy number X isolates and high copy number S isolates displayed conservation of PIRs. Table 4 lists conserved insertion positions in LAM, S and X isolates. In an earlier study, Rv1755c was suggested to be a hotspot^22^ and Rv1755c containing IS*6110* was found in EAI6 and S isolates but not in any other L1 and L4 isolates.

The DR region was the only region where the IS element is found in almost all isolates. The results of lineage specific PIRs indicate a lack of sequence specificity or general preferential location similar to that suggested by Thorne *et al*^24^. Since it is difficult to find IS insertions in exactly the same nucleotide position in two different isolates, in this study a window of 100 nucleotides was used to mark conservation. The exact site of conservation varied in different cases, for example, all L2 isolates had an insertion in 1592–1594 whereas, region 888700–888900 was found with different insertion locations in almost all lineages.

### IS*6110* in intragenic region

Insertion of a number of IS*6110* was observed within coding regions of genes (9750 out of 19366 insertion sites). It appears that there is a low degree of specificity in terms of selection of genes as IS*6110* was found in 368 genes. DAVID enrichment analysis^42^ using gene ontology terms was performed in order to identify any functional specificity associated with these insertions. Plasma membrane, Cell membrane, Glycerophospholipid metabolism, Mycobacterial PPE protein, Mycobacterial pentapeptide repeat, naphthalene and anthracene degradation were some of the top ranked terms (p-value < 0.0001). Genes were also classified using COG and the results are shown in supplementary figure 2. Genes that map to the class cell motility are highly represented, followed by signal transduction, defense, replication-recombination-repair mechanisms and cell wall/membrane/envelope biogenesis. The element was not seen in genes that map to COG classes intracellular trafficking, secretion and vesicular transport. Tuberculist^43^ classification of *M. tuberculosis* genes was also used and the results showed high level of insertions in repetitive sequences and gene families, such as PE/PPE family proteins, IS and phages, and regulatory proteins. This study suggests that genes involved in Information pathways and virulence do not normally host these elements due to their importance in pathogenesis. Predominant IS*6110* insertion and SNPs^44^ in PE/PPE family genes signifies increased fitness or relaxed selection in this class of proteins.

### IS*6110* as a mobile promoter

IS*6110* carries a promoter element and can activate a gene when inserted upstream of it’s coding sequence. In order to identify elements that are likely to be close to a functional gene, insertions at <400 bp^21^ from transcription start site and in the same orientation as the downstream gene were located. It is expected that expression of some of these genes may be regulated by the element. A total of 4796 such positions were identified in 1377 isolates. Overall 178 unique genes were found that carried IS*6110* insertion immediately in the upstream region. Twenty seven of these genes are thought to be essential. These genes belong to mainly three categories, transposase, oxidoreductase and PE-PPE family proteins. *TrpD* and Rv1668c (ABC transporter) are the exceptions. *TrpD* is essential for survival of bacteria in activated macrophages and during lung colonization^45^. As a consequence, up-regulation of this gene is beneficial for the organism. Several other insertions upstream of the *phoP* gene at 131, 46, 94 and 41 bp upstream in L2, L3, L1 and L5 isolates respectively was obseerved. Previously reported multi-drug resistant L2 isolates also displayed an insertion 75 bp upstream of the *phoP* gene. Transcriptional regulators, other than *phoP* are also the target of IS*6110* insertions. For example, the promoter regions of Rv0894 (L2), Rv1033c/*trcR*, Rv3124 (L3), Rv3246c/*mtrA* and Rv3334 (L4) were also found to have IS*6110* elements. Many insertions in the promoter regions detected by us have already been reported such as, the promoter regions of Rv2353, Rv2280, Rv3427 (38 bp upstream in 300 L2, 2 L3 and 3 L4 isolates) and Rv3018 (308 bp upstream of 232 L2 isolates)^34^,^46^ validating the method used here. ESAT-6 related proteins play an important role in mycobacterial virulence. Several Esat-6 related genes, such as *esxJ*, *esxQ*, *esxR* and *esxS* were found with IS*6110* in their promoter regions in a number of isolates.

### Identification of IS*6110* mediated LSPs

IS elements are known for their ability to cause LSPs due to recombination of neighboring copies^47^. Experimental methods to identify IS*6110*-mediated recombination events look for IS elements in the absence of surrounding direct repeats^21^. Deletions and inversions from 1377 isolates were identified and events where neighboring regions showed the presence of IS*6110* were filtered. In total, 2414 such events were identified from all isolates. Polymorphism with respect to the length of sequence involved was also observed in the conserved polymorphic sites. Table 5 lists some of the common variants found in at least 10 isolates. Events noticed in <10 isolates are not included in the table as these events may be sporadic and are unlikely to display any pattern. No evidence for lineage specific LSPs (present in most of the isolates of one lineage but absent in others) was found. Mostly two types of events were identified: i) deletion of a neighboring region of IS*6110* and ii) inversion of a region containing IS*6110* and flanking regions. Moreover, the number of IS*6110*-mediated LSP in a lineage is positively correlated with the mean copy number of IS*6110* (Pearson correlation coefficient, R = 0.811, P < 0.05). It is expected that isolates that show a different distribution of IS elements may display different genomic features. For example, a higher number of IS-mediated recombination events may have more chance of occurring in isolates with high copy number of IS*6110*. More recombination events would also cause genomic changes that may alter phenotype of the isolates. The presence of an upper limit in copy number is also either due to an active process that inhibits further transposition or due to secondary loss of transposed copies by recombination of neighboring elements.

### IS*6110* based global phylogeny of *M. tuberculosis*

**Figure 2:**
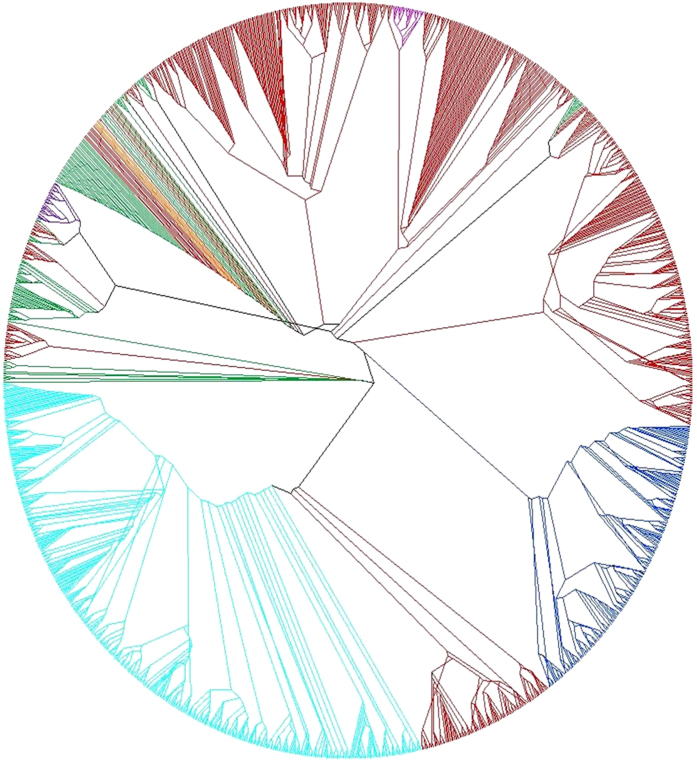
Global phylogeny of 1377 *M.* *tuberculosis* isolates based on IS*6110* insertion sites. Different colors represent isolates from different lineages. L1: green; L2: cyan; L3: blue; L4: red; L5: purple; L6: violet; L7: orange.

IS*6110* insertion sites in all 1377 isolates were used to construct a phylogenetic tree. Detailed methodology is described in “methods”. The results show that five of seven lineages (L2: cyan, L3: blue, L5: purple, L6: violet, L7: orange) map to distinct clusters in the tree (Fig. 2). Low copy number isolates of L1 (green) and L4 (red) cluster with L7 due to insertion of IS*6110* in DR region. As expected, isolates of L4 were distributed in different clusters and not part of a separate cluster. Therefore, only L4 isolates have been analyzed (supplementary figure 3). Isolates belonging to spoligotypes LAM, X and S were found to be in distinct clusters, whereas those with H and T spoligotype patterns cluster together. It is likely that some of the results may be due to wrong assignment of some of these spoligotypes (PolyTB database^48^) due to convergence^49^. Similarities and differences with SNP based phylogenetic tree were clearly visible. A SNP based phylogenetic tree (supplementary figure 4) clearly separated out each of the lineages unlike that of IS*6110* insertion sites. Moreover, the former places the two West African lineages in close proximity, in contrast to IS*6110* based tree which maps these at a distance. Larger set of data points in SNP based tree is one of the main reasons for better resolution. Though each lineage shows a clear evidence of independent evolution of IS*6110* in the Mycobacterial genome, several reasons, such as recombination of neighboring elements, insertion in a transcriptionally activated region or transcriptional control may generate homoplasy or convergent evolution which is otherwise rare in *M. tuberculosis* complex^12^.

Attempts to relate phenotype (such as drug resistance) to IS insertion did not give clear results (absence of PIR). Though there are reports to indicate that location/site of insertion can alter expression of important genes, no difference was observed when drug sensitive and drug resistant isolates were compared (Two sided paired t-test with fraction of isolates positive for IS*6110* in each genomic bin; t = 1.1196, df = 288, p = 0.263). However, this study identified insertions in some of the essential genes^29^ in one or more drug-resistant isolates, but not in drug-sensitive ones. Some of these genes are Rv2026c, Rv2283 and Rv2808 in L2 and Rv3398 in L4.

## Conclusion

This study provides novel insights into IS*6110* based mycobacterial genome evolution using the largest data size so far reported. Overall, the study identifies IS*6110* based molecular markers for strain typing as well as *M. tuberculosis* classification and can be used for lineage classification. It highlights intra and inter-lineage variations with respect to element copy number, preferential insertion regions and possible effect on genome evolution due to the presence of these elements. In conclusion these results show the importance of global comprehensive analysis of IS*6110* insertion with respect to epidemiological and evolutionary perspective of *M. tuberculosis* genome.

## Method

The overall pipeline of IS-element identification is described in supplementary figure 5 following Das *et al.*^40^. A split-read approach was used for identification of IS-element insertion sites in *M. tuberculosis* genome. Initially NGS reads that overlapped with IS-elements as well as flanking regions were identified by aligning reads with a reference IS*6110* using a local alignment scheme. Local alignment allows soft-clipped reads to align partially with the reference. Reads containing at least 10 bp sequence from flanking regions were considered (overlap with the reference depends on the read length, minimum overlap depends on minimum score threshold of aligner which in turn depends on read length whereas maximum overlap was read length - 10). 5′ fragments and 3′ fragments were trimmed from original NGS reads (supplementary figure 6) and were then clustered separately so that reads coming from a genomic region should fall in the same cluster. To get rid of sequencing errors, one consensus sequence was obtained from each cluster by a local assembly of the sequences in one cluster. Let, m be the number of clusters generated from 5′ fragments and n be the number of clusters generated from 3′ fragments. Copy number of IS*6110* was estimated by c = min (m,n) and supported by read depth analysis in respect to average read depth of the genome. m and n consensus sequences were again aligned independently with the reference genome. The method is described in a flowchart in supplementary figure 7. Alignment locations and strand information of 5′–3′ pairs were taken into consideration for finding IS*6110* insertion with respect to reference genome. Overlapping alignment locations were used to identify direct repeats generated upon insertion of IS*6110*. Moreover, a distance of approximately 1355 bp suggested presence of IS*6110* in the same locus as that of the reference. Unpaired alignments (as per above criteria) were treated separately for identification of IS-mediated LSPs (deletion and inversion).

The tools along with PERL codes used for implementation and automation are Bowtie2^50^ (for local alignment of reads), BlastClust in BLAST^51^ suite (for clustering), CAP3^52^ (for assembly of fragmented reads in each cluster) and BLASTn for alignment of consensus sequences to the reference genome. Parameters for these software are available as supplementary information. Deletions and inversions were predicted by Pindel^53^. Only deletions and inversions larger than 20 bp were considered. SNP based phylogenetic tree was constructed using NexABP^54^. Jaccard distance^55^ was used to calculate distance matrix for IS*6110* based phylogenetic tree construction. All available insertion sites in different isolates were used to construct binary strings for each isolate. Binary strings were generated depending upon the presence or absence of IS element in a 100 bp domain. These strings were then compared to calculate isolate-vs-isolate distance. Phylogenetic trees were constructed by Neighbor-joining algorithm in Phylip^56^. Trees were then visualized using Dendroscope^57^.

### Datasets

NGS datasets were downloaded from European Nucleotide Archive, EMBL. Accession numbers are listed in supplementary dataset. Spoligotype and lineage information were obtained from PolyTB database^48^.

## Additional Information

**How to cite this article**: Roychowdhury, T. *et al.* Analysis of IS*6110* insertion sites provide a glimpse into genome evolution of *Mycobacterium tuberculosis. Sci. Rep.* **5**, 12567; doi: 10.1038/srep12567 (2015).

## Supplementary Material

Supplementary Information

Supplementary Information

## Figures and Tables

**Table 1:** *M. tuberculosis* isolates used for this analysis.

**Lineage**	**Number of isolates**
Indo-Oceanic (L1)	116
East Asian (L2)	375
Indian-East African (L3)	182
Euro-American (L4)	666
West African I (L5)	18
West African II (L6)	16
Ethiopian (L7)	4

**Table 2:** Lineage specific preferential insertion regions of IS*6110.*

**L2 Domains**	**Percentage of L2 isolates (threshold >80%)**	**Percentage of other isolates (threshold <10%)**	**Annotation**
1500–1600	97.84	0.75	Intergenic (Rv0001-Rv0002)
1262900–1263000	84.68	0.46	Rv1135c (PPE16)
1543900–1544000	95.93	0.46	Rv1371 (hypothetical)
1998600–1998700	94.25	4.60	Intergenic(Rv1765-Rv1765A)
2263600–2263700	94.01	0.46	Rv2016 (hypothetical)
2634000–2634100	86.12	7.04	Rv2352c (PPE38)
3378500–3378600	91.62	0.28	Intergenic (Rv3018-Rv3019)
3549100–3549200	81.10	3.00	Intergenic (Rv3179-Rv3180)
3797800–3797900	93.30	0.84	Rv3383c (idsB)
3844600–3844700	88.51	0.46	Rv3427c (transposase)
**L3 Domains**	**Percentage of L3 isolates (threshold >80%)**	**Percentage of other isolates (threshold <10%)**	**Annotation**
475200–475300	98.48	0.46	Rv0395 (hypothetical)
850000–850100	92.92	0.77	Rv0755c (PPE12)
1694600–1694700	98.98	0	Rv1504c (hypothetical)
2166300–2166400	84.34	0.07	Rv1917c (PPE34)
3048400–3048500	92.92	0.15	Rv2735c (hypothetical)
4320100–4320200	98.48	0.07	Intergenic (Rv3845-Rv3846)
**L5 Domains**	**Percentage of L5 isolates (threshold >80%)**	**Percentage of other isolates (threshold <10%)**	**Annotation**
1075900–1076000	92.30	4.73	Rv0963c (hypothetical)
1907000–1907100	92.30	0.48	Rv1682 (coiled-coil str. Pr.)
2555700–2555800	96.15	0.82	Rv2282c (LysR family transcriptional regulator)
4185700–4185800	96.15	0	Rv3734c (hypothetical)
**L6 Domains**	**Percentage of L6 isolates (threshold >80%)**	**Percentage of other isolates (threshold <10%)**	**Annotation**
1300100–1300200	95.65	0	Rv1169c (PE11)
3709600–3709700	95.65	0.54	Rv3323c (moaX)

**Table 3:** Spoligotype specific preferential insertion regions of IS*6110* in L1 isolates.

**EAI2 (Manila, Nonthaburi)**	**Percentage of EAI2 isolates (threshold >75%)**	**Percentage of other L1 (threshold <20%)**	**Annotation**
888800–888900	100	2.46	Intergenic (Rv0794c-Rv0795)
932100–932200	88.88	0	Intergenic (Rv0835-Rv0836c)
1721300–1721400	100	0	Rv1526c (glycosyltransferase)
1998600–1998700	88.88	0	Intergenic (Rv1765c-Rv1765A)
2038800–2038900	88.88	0	Intergenic (Rv1798-Rv1799)
**EAI6 (BGD1)**	**Percentage of EAI6 isolates (threshold >75%)**	**Percentage of other L1 (threshold <20%)**	**Annotation**
888700–888800	97.87	3.92	Intergenic (Rv0794c-Rv0795)
1880600–1880700	97.87	3.92	Rv1661 (pks7)
1987600–1987700	97.87	3.92	Rv1755c (plcD)
2559500–2559600	97.87	3.92	Rv2286c (hypothetical)
2627900–2628000	97.87	3.92	Rv2349c (plcC)
3030100–3030200	97.87	3.92	Rv2717c (hypothetical)
3096500–3096600	97.87	3.92	Rv2787 (hypothetical)
3491500–3491600	97.87	3.92	Rv3125c (PPE49)

**Table 4:** Spoligotype specific preferential insertion regions of IS*6110* in L4 isolates.

**LAM**	**Percentage of LAM isolates (threshold >75%)**	**Percentage of other L4 (threshold <20%)**	**Annotation**
932200–932300	86.89	3.25	Intergenic (Rv0835-Rv0836c)
1481500–1481600	76.21	0.25	Rv1319c (adenylate cyclase)
3480300–3480400	88.34	3.5	Rv3113 (phosphatase)
**S**	**Percentage of S isolates (threshold >75%)**	**Percentage of other L4 (threshold <20%)**	**Annotation**
80400–80500	100	0.16	Intergenic (Rv0071-Rv0072)
888900–889000	100	3.37	Intergenic (Rv0794c-Rv0795)
1889000–1889100	100	0.16	Rv1664 (pks9)
1987400–1987500	100	3.37	Rv1755c (plcD)
2166500–2166600	100	1.01	Rv1917c (PPE34)
**X**	**Percentage of X isolates (threshold >75%)**	**Percentage of other L4 (threshold <20%)**	**Annotation**
483200–483300	100	16.07	Rv0402c (mmpL1); Rv0403c (mmpS1)

**Table 5:** Probable IS*6110* mediated large sequence polymorphisms in different isolates.

**Co-ordinate and type of large sequence polymorphism (D: deletion, I: inversion)**	**Location of neighboring IS*****6110*** **In H37RV**	**Lineage (Number of isolates With the same polymorphism)**
888755–889021 (D)	889021–890375	L4(15)
888762–889021 (D)		L4(19)
888785–889021 (D)		L1(65), L2(278), L3(4), L4(9)
1543306–1543969 (D)	1541952–1543306	L2(11)
1989057–1989078 (D)	1987703–1989057	L3(126)
1989057–1989080 (D)		L4(46)
1996100–1998623 (I)	1996100–1997455	L2(352), L3(1), L4(2)
1996100–1998750 (I)		L3(23)
1996100–1998792 (I)		L2(14)
1996100–1998810 (I)		L4(146)
1996100–1998838 (I)		L4(44)
1997455–1998658 (D)		L4(12)
2365413–2367206 (I)	2365414–2366768	L2(14), L4(2)
2366766–2367209 (D)		L2(12)
2628292–2635577 (D)	2635577–2636931	L3(36)
2634026–2635577 (D)		L2(22)
2635041–2635577 (D)		L2(14)
2635577–2636956 (I)		L3(44)
2636931–2636955 (D)		L2(12), L3(43)
2636931–2639361 (D)		L4(14)
3121878–3122030 (D)	3120523–3121897	L1(41)
3121878–3121986 (D)		L3(48)
3549196–3551230 (D)	3551230–3552584	L2(18)
