# Supplementary material for: Analysis of IS6110 insertion sites provide a glimpse into genome evolution of Mycobacterium tuberculosis
Source: Sci Rep. 2015 Jul 28;5:12567. doi: 10.1038/srep12567 (PMC4517164; doi:10.1038/srep12567)
Supplement: Supplementary Information [file srep12567-s1.pdf]

# Analysis of IS6110 insertion sites provide a glimpse into genome evolution of *Mycobacterium tuberculosis*

Tanmoy Roychowdhury[1], Saurav Mandal[1], Alok Bhattacharya[1,2] *

[1]School of Computational and Integrative Sciences, Jawaharlal Nehru University, New Delhi

[2]School of Life Sciences, Jawaharlal Nehru University, New Delhi

**Supplementary Figure**

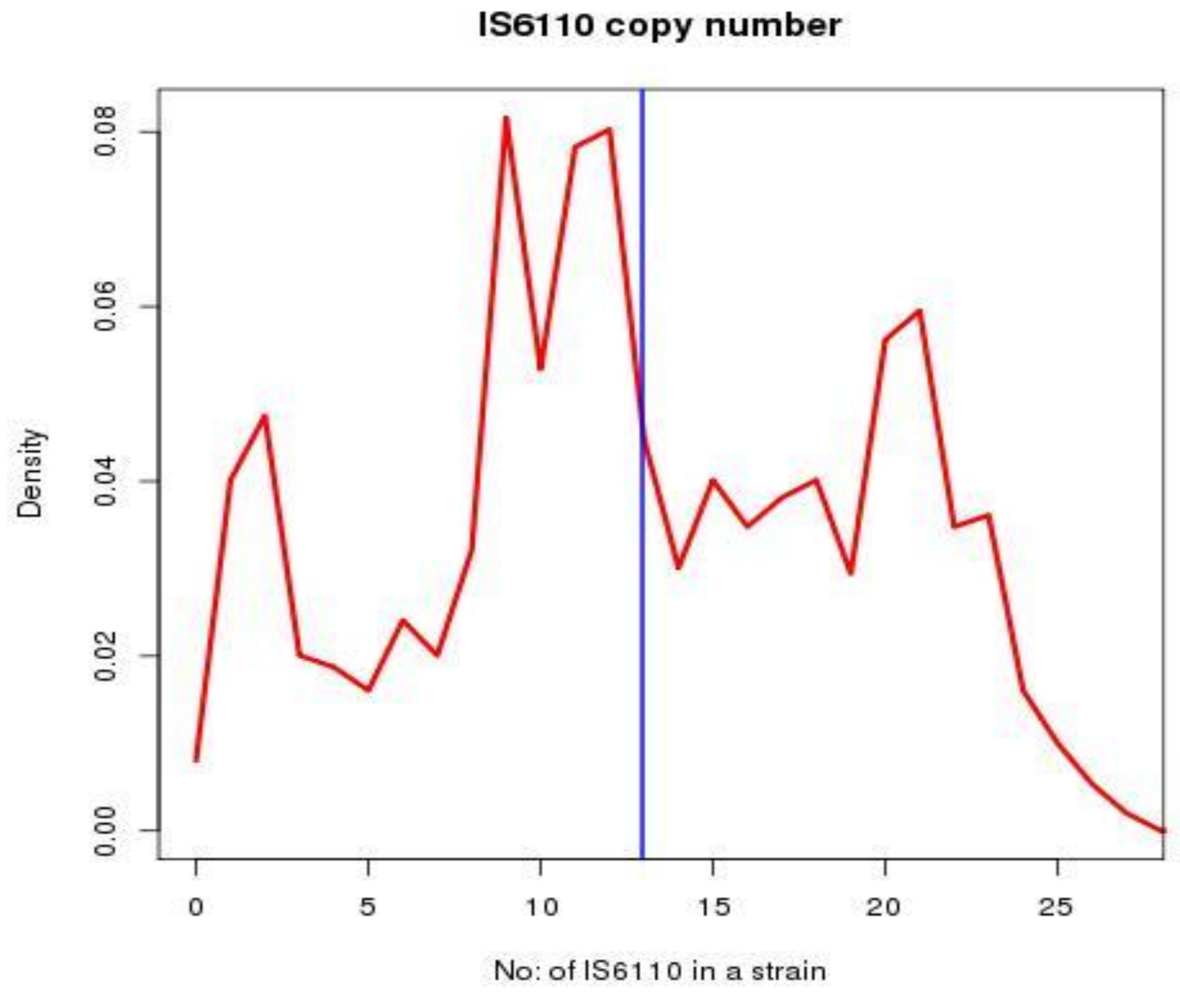


**Supplementary figure 1:** Distribution of IS6110 copy number in 1377 strains. The blue line signifies mean copy number

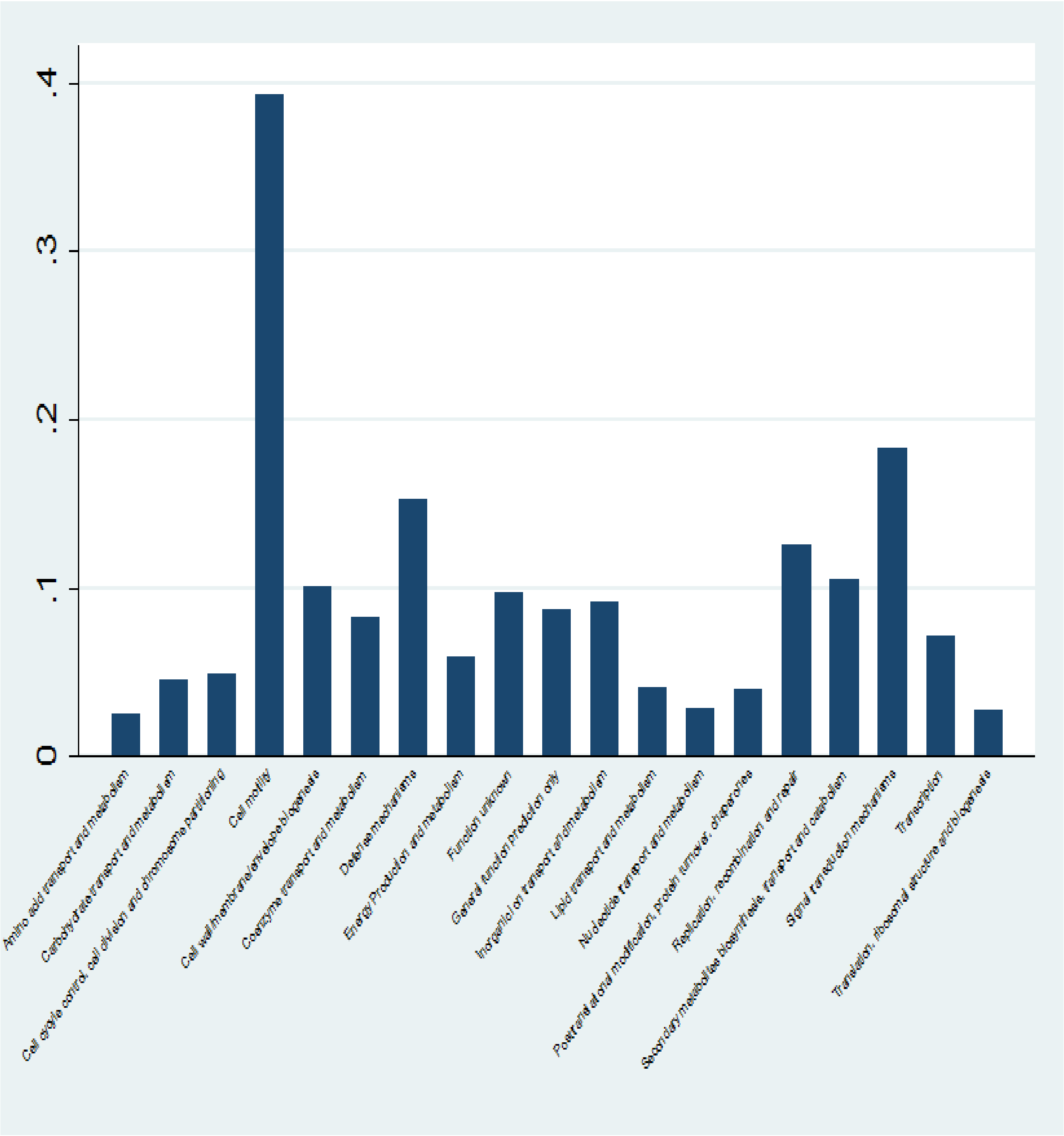


**Supplementary figure 2:** COG based classification of genes where IS6110 insertion been identified in at least one strain.

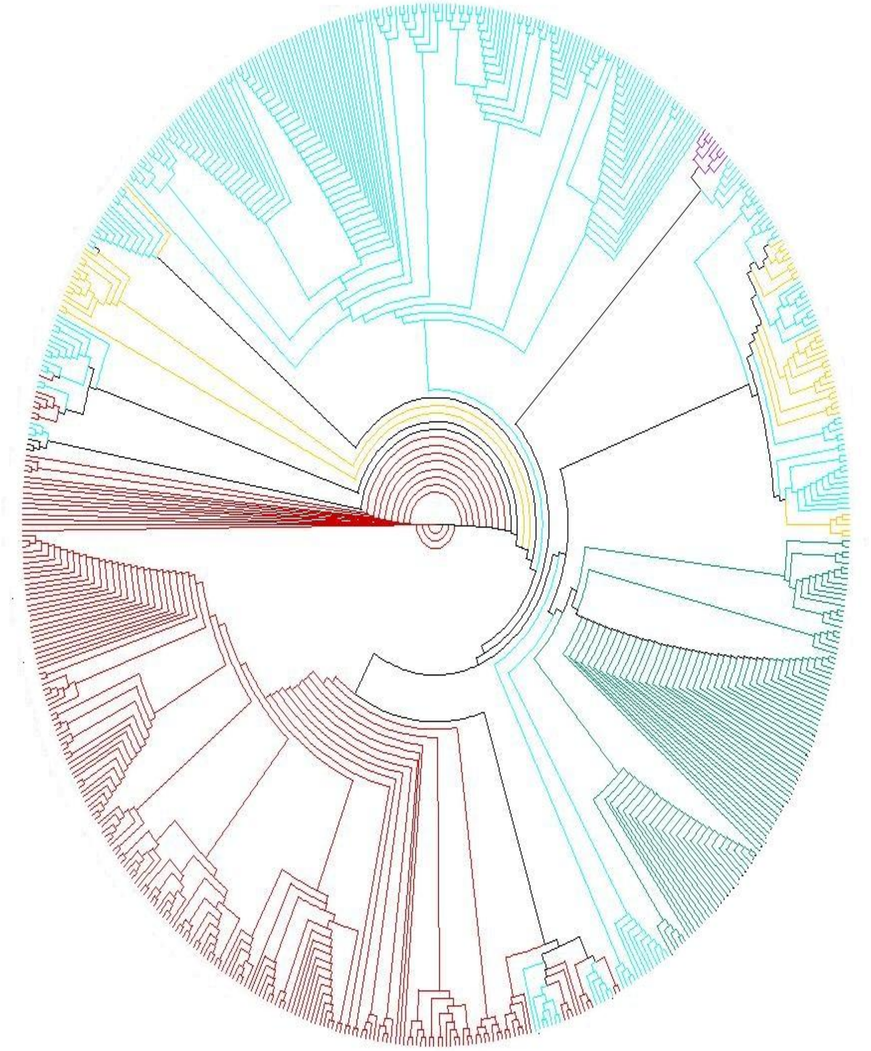

**Supplementary figure 3:** Phylogeny of *M.tuberculosis* L4 strains based on IS6110 insertion sites. Different colors represent strains from different spoligotyping classes. LAM: red; X: green; T: cyan; H: yellow; S: purple

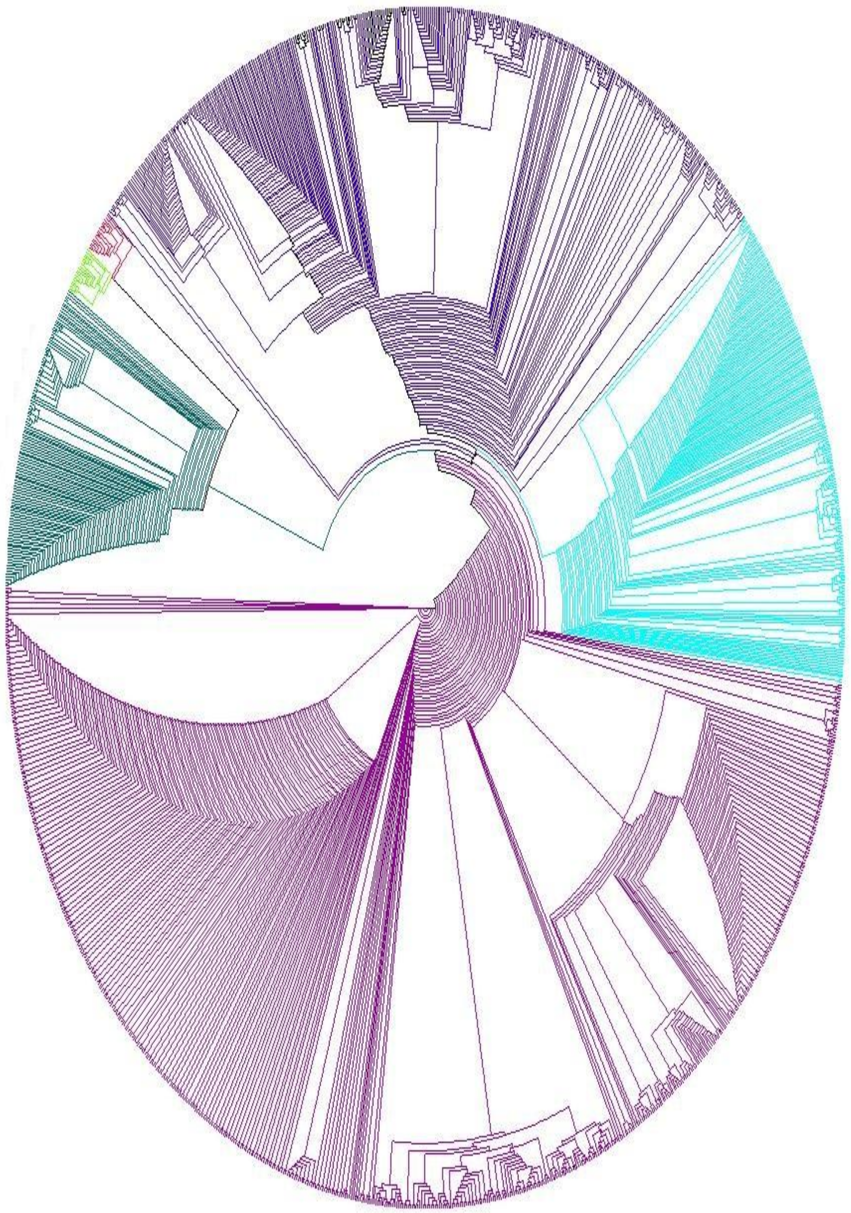

**Supplementary figure 4:** Global phylogeny of *M. tuberculosis* isolates based on SNP. Different colors represent isolates from different lineages. L1: Green; L2: Blue; L3:Cyan; L4: Purple; L5: Light green; L6: Red

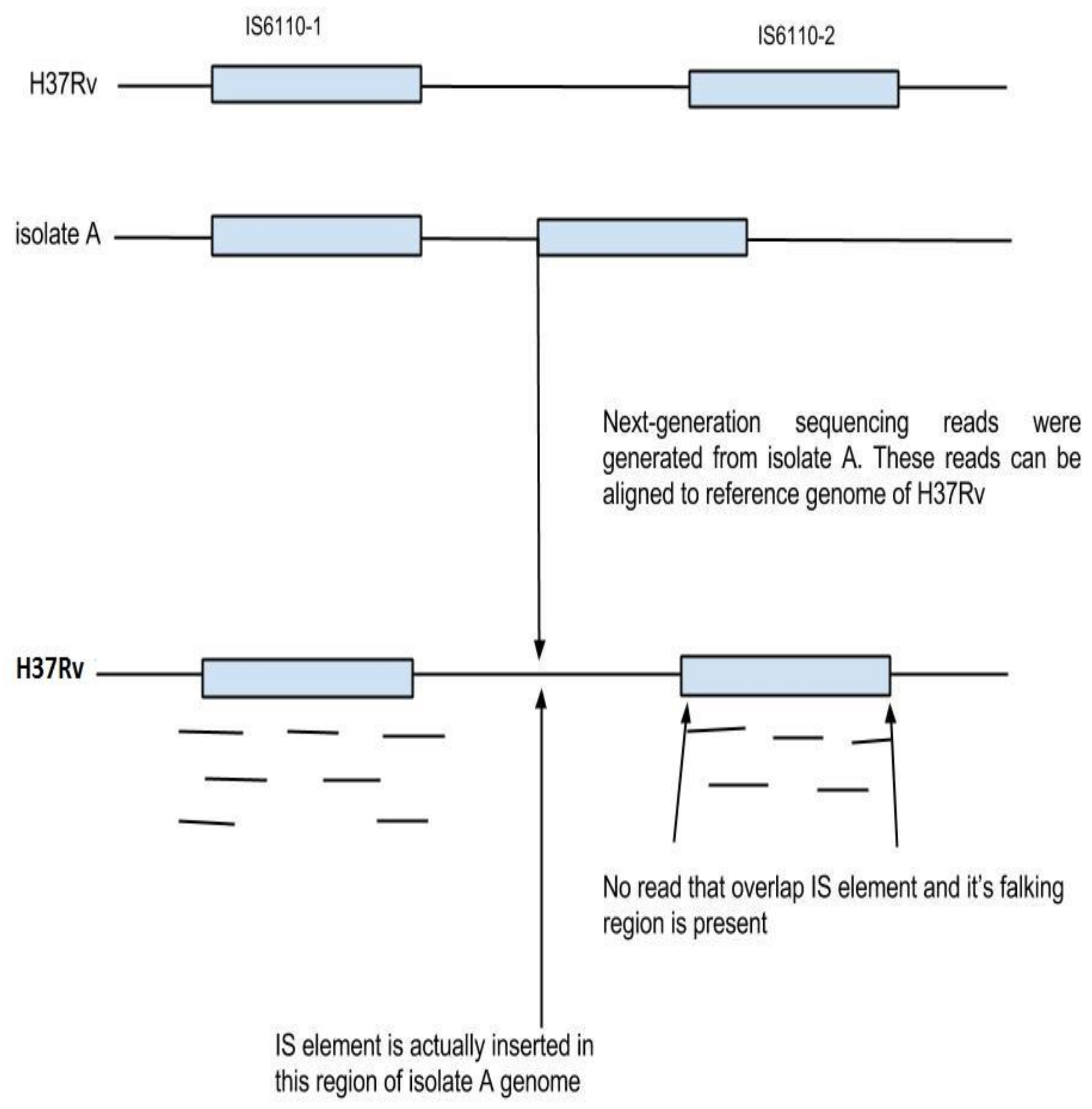


**Supplementary figure 5:** Comparison of IS6110 location in two Mycobacterial strain.

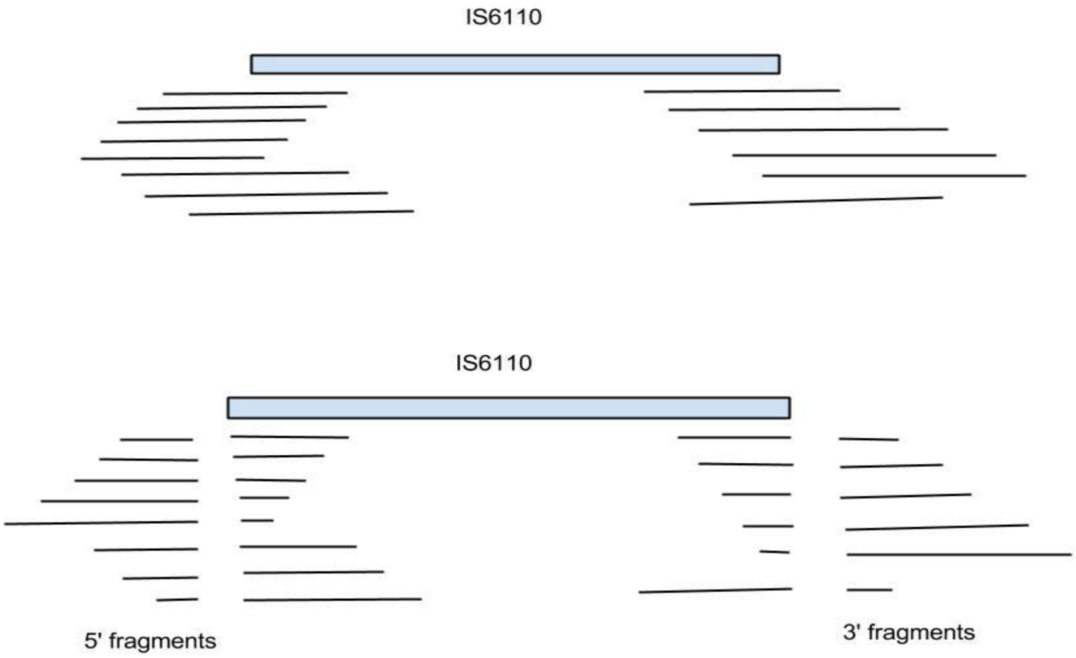


**Supplementary figure 6:** Local alignment of NGS reads against IS6110 sequence

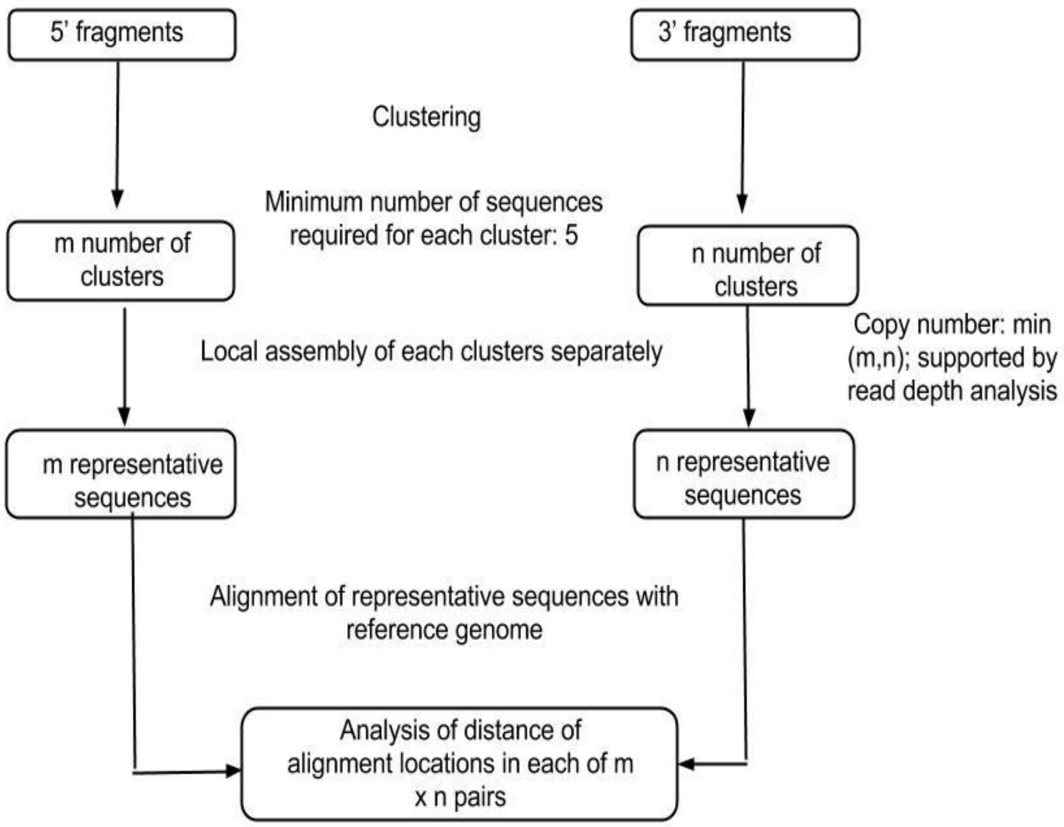


**Supplementary figure 7:** Flowchart for identification of IS element insertion locations in reference genome.

## Supplementary Table

**Supplementary table 1:** Lineage wise Copy number mean and standard deviation

| Lineage | Mean | Standard deviation |
|---|---|---|
| L1 | 6.5 | 5.34 |
| L2 | 20.24 | 3.64 |
| L3 | 16.13 | 2.98 |
| L4 | 9.25 | 4.32 |
| L5 | 12.07 | 1.44 |
| L6 | 4.04 | 1.29 |
| L7 | 1 | 0 |

**Supplementary table 2:** Spoligotype wise (L1 and L4 isolates) Copy number mean and standard deviation

| Spoligotype | Mean | Standard deviation |
|---|---|---|
| EAI1 | 2.33 | 1.79 |
| EAI2 | 11.33 | 2.06 |
| EAI3 | 1.25 | 0.45 |
| EAI4 | 1 | 0 |
| EAI5 | 4.25 | 4.67 |
| EAI6 | 12.33 | 1.13 |
| T | 10.01 | 4.32 |
| X | 3.58 | 1.79 |
| LAM | 10.56 | 2.99 |
| H | 10.42 | 3.75 |
| S | 11.28 | 1.25 |

**Supplementary Information**

**Command and parameters for component software:**

- bowtie2-build IS6110_ref.txt IS6110
- bowtie2 --local -x IS6110  -U file.fq  -S temp.sam
- blastclust -p F -L 1 -b F -a 2 -W 10 -i 5’frag.txt -o 5’frag_clust.txt
- blastclust -p F -L 1 -b F -a 2 -W 10 -i 3’frag.txt -o 3’frag_clust.txt
- cap3  seq_in_one_cluster.txt -i 30  -j 31  -o 18  -s 300
- makeblastdb -in mtbh37rv.fasta -dbtype 'nucl' -out mtbh37rv
- blastn -db mtbh37rv -query cluster_rep_consensus_generated_by_cap3.txt -out location_in_Rv.txt -evalue 0.00001 -outfmt 6
